# Orexin in the anxiety spectrum: association of a *HCRTR1* polymorphism with panic disorder/agoraphobia, CBT treatment response and fear-related intermediate phenotypes

**DOI:** 10.1038/s41398-019-0415-8

**Published:** 2019-02-04

**Authors:** Michael G. Gottschalk, Jan Richter, Christiane Ziegler, Miriam A. Schiele, Julia Mann, Maximilian J. Geiger, Christoph Schartner, György A. Homola, Georg W. Alpers, Christian Büchel, Lydia Fehm, Thomas Fydrich, Alexander L. Gerlach, Andrew T. Gloster, Sylvia Helbig-Lang, Raffael Kalisch, Tilo Kircher, Thomas Lang, Tina B. Lonsdorf, Christiane A. Pané-Farré, Andreas Ströhle, Heike Weber, Peter Zwanzger, Volker Arolt, Marcel Romanos, Hans-Ulrich Wittchen, Alfons Hamm, Paul Pauli, Andreas Reif, Jürgen Deckert, Susanne Neufang, Michael Höfler, Katharina Domschke

**Affiliations:** 1 Department of Psychiatry and Psychotherapy, Medical Center – University of Freiburg, Faculty of Medicine, University of Freiburg, Freiburg, Germany; 2 Department of Psychiatry, Psychosomatics and Psychotherapy, Center of Mental Health, University Hospital of Würzburg, Würzburg, Germany; 3 Department of Biological and Clinical Psychology/Psychotherapy, University of Greifswald, Greifswald, Germany; 4 Epilepsy Center, Medical Center - University of Freiburg, Faculty of Medicine, University of Freiburg, Freiburg, Germany; 5 Department of Physiology, University of California San Francisco, San Francisco, CA USA; 6 Department of Neuroradiology, University of Würzburg, Würzburg, Germany; 7 Department of Psychology, School of Social Sciences, University of Mannheim, Mannheim, Germany; 8 Department of Systems Neuroscience, University Medical Center Hamburg-Eppendorf, Hamburg, Germany; 9 Department of Psychology, Humboldt University, Berlin, Germany; 10 Department of Clinical Psychology and Psychotherapy, University of Cologne, Cologne, Germany; 11 Department of Psychology, Institute of Clinical Psychology and Psychotherapy, Technische Universität Dresden, Dresden, Germany; 12 Division of Clinical Psychology and Intervention Science, University of Basel, Basel, Switzerland; 13 Department of Psychology and Psychotherapy, University of Hamburg, Hamburg, Germany; 14 Neuroimaging Center (NIC) und Deutsches Resilienz-Zentrum (DRZ), Johannes Gutenberg University Medical Center, Mainz, Germany; 15 Department of Psychiatry and Psychotherapy, University of Marburg, Marburg, Germany; 16 Christoph-Dornier-Foundation for Clinical Psychology, Bremen, Germany; 17 Department of Psychiatry and Psychotherapy, Campus Charité Mitte, Charité – Universitätsmedizin Berlin, corporate member of the Freie Universität Berlin, Humboldt-Universität zu Berlin, and Berlin Institute of Health, Berlin, Germany; 18 Department of Psychiatry, Psychosomatic Medicine and Psychotherapy, University Hospital of Frankfurt, Frankfurt, Germany; 19 Department of Psychiatry and Psychotherapy, University Hospital of Münster, Münster, Germany; 20 kbo-Inn-Salzach-Hospital, Wasserburg, Germany; 21 Department of Psychiatry und Psychotherapy, Ludwig Maximilians University, Munich, Germany; 22 Department of Child and Adolescent Psychiatry, Psychosomatics and Psychotherapy, Center of Mental Health, University Hospital of Würzburg, Würzburg, Germany; 23 Department of Psychology, Center of Mental Health, University of Würzburg, Würzburg, Germany; 24 Department of Psychiatry and Psychotherapy, Medical Faculty Heinrich-Heine University, Duesseldorf, Germany; 25 Center for NeuroModulation, Faculty of Medicine, University of Freiburg, Freiburg, Germany

## Abstract

Preclinical studies point to a pivotal role of the orexin 1 (OX_1_) receptor in arousal and fear learning and therefore suggest the *HCRTR1* gene as a prime candidate in panic disorder (PD) with/without agoraphobia (AG), PD/AG treatment response, and PD/AG-related intermediate phenotypes. Here, a multilevel approach was applied to test the non-synonymous *HCRTR1* C/T Ile408Val gene variant (rs2271933) for association with PD/AG in two independent case-control samples (total *n* = 613 cases, 1839 healthy subjects), as an outcome predictor of a six-weeks exposure-based cognitive behavioral therapy (CBT) in PD/AG patients (*n* = 189), as well as with respect to agoraphobic cognitions (ACQ) (*n* = 483 patients, *n* = 2382 healthy subjects), fMRI alerting network activation in healthy subjects (*n* = 94), and a behavioral avoidance task in PD/AG pre- and post-CBT (*n* = 271). The *HCRTR1* rs2271933 T allele was associated with PD/AG in both samples independently, and in their meta-analysis (*p* = 4.2 × 10^−7^), particularly in the female subsample (*p* = 9.8 × 10^−9^). T allele carriers displayed a significantly poorer CBT outcome (e.g., Hamilton anxiety rating scale: *p* = 7.5 × 10^−4^). The T allele count was linked to higher ACQ sores in PD/AG and healthy subjects, decreased inferior frontal gyrus and increased locus coeruleus activation in the alerting network. Finally, the T allele count was associated with increased pre-CBT exposure avoidance and autonomic arousal as well as decreased post-CBT improvement. In sum, the present results provide converging evidence for an involvement of *HCRTR1* gene variation in the etiology of PD/AG and PD/AG-related traits as well as treatment response to CBT, supporting future therapeutic approaches targeting the orexin-related arousal system.

## Introduction

The general term orexin (or hypocretin) refers to the two known hypothalamic neuropeptides comprised in the orexin class, namely orexin-A and -B (hypocretin 1/2)^1^. The orexin system has been implicated in the maintenance of arousal, wakefulness, and vigilance as well as in influencing motivated behaviors such as emotional responses, reward seeking and feeding^2^.

The action of orexin-A and -B is mediated by two subtypes of G-protein coupled receptors, the orexin 1 (OX_1_) receptor and the orexin 2 (OX_2_) receptor^3,4^. While both receptors are co-expressed in the dorsal raphe nuclei and ventral tegmental area, the OX_1_ and OX_2_ receptors display characteristic anatomical distributions, suggesting distinct physiological functions based on specific neuronal signaling pathways: the OX_1_ receptor is primarily expressed in the locus coeruleus (LC), laterodorsal and pedunculopontine tegmental nucleus, while the OX_2_ receptor is located in the arcuate and tuberomammillary nucleus^5^. In addition, site-specific exclusive expression of the OX_1_ receptor has been reported in the cingulate cortex, CA1/2 region of the hippocampus, bed nucleus of the stria terminalis and amygdala^5^. Nonetheless, it should be kept in mind that gene expression information is ultimately limited by mRNA detection and antibody staining sensitivity and specificity.

Besides this abundance of the OX_1_ receptor in circuity engaged during fear^6^, a pivotal role of the OX_1_ receptor in the pathogenesis of hyperarousal- and panic-related anxiety has been suggested by both preclinical and clinical studies:^7^ In a rodent model, the orexin system has been shown to modulate the formation and expression of fear memory via noradrenergic neurons in the LC expressing the OX_1_ receptor, with *Hcrtr1*(−/−) mice displaying impaired freezing responses in both cued and contextual fear-conditioning paradigms^8^. Additionally, blocking orexin activity in the noradrenergic LC has been reported to reduce fear learning in a comparable fear conditioning paradigm^9^. Accordingly, selective OX_1_ receptor antagonism has been shown to attenuate anxiety-related behavior in the open field and social interaction test and to reduce neural responses in the central nucleus of the amygdala, bed nucleus of the stria terminalis, periaqueductal gray and in the rostroventrolateral medulla^10^. Correspondingly, pre-treatment with an OX_1_ receptor antagonist attenuated sodium lactate-induced anxiety-related behavior, locomotor, and cardioexcitatory responses and even worked in a panic-prone strain of rats in a comparable fashion to alprazolam^11^. In a similar vein, pre-treatment with an OX_1_ receptor antagonist attenuated hypercapnia-induced panic-related behavior and hypertension^12^. Furthermore, systemic OX_1_ receptor blockage and site-specific blockage in the amygdala have been demonstrated to support the extinction of aversive memories, facilitating the consolidation of cue- and context-dependent fear extinction, potentially via increased infralimbic medial-prefrontal cortex activity^13^. Translating preclinical research to potential clinical applications, human subjects reporting panic-associated symptoms have been found to display elevated qualitative levels of orexin in the cerebrospinal fluid (CSF) compared to subjects without panic-like anxiety^11^. Additionally, chronic treatment with sertraline, a first-line anti-panic drug, has been observed to reduce orexin levels in the CSF of depressed patients^14^.

In sum, given the large body of evidence supporting a key role of particularly the OX_1_ receptor in hyperarousal- and panic-related fear reactions, the gene coding for the OX_1_ receptor (*HCRTR1*; chromosome 1p35.2) — containing a potentially functionally relevant C/T single nucleotide polymorphism (SNP) (rs2271933) in exon 7 leading to an amino acid exchange from isoleucine to valine (Ile408Val)^15–18^ — is suggested as a prime candidate regarding these phenotypes. Thus, in a multilevel approach, we examined the association between *HCRTR1* rs2271933 genotype and panic disorder in two independent samples of patients with panic disorder (PD) with and without agoraphobia (AG) and healthy controls. Within these samples, we explored the possibility of predicting treatment response according to clinical outcome measures based on *HCRTR1* rs2271933 genotype in a controlled and randomized exposure-based cognitive-behavioral therapy (CBT) trial. Subsequently, we tested for an association between *HCRTR1* rs2271933 genotype and agoraphobic cognitions as measured by the Agoraphobic Cognitions Questionnaire (ACQ) in both PD/AG patients and healthy subjects. Activation in the alerting network as elicited by the attention network task (ANT) — comprising the LC as well as right lateralized fronto-parietal regions particularly important for anxiety-related arousal regulation^19,20^ — was investigated dependent on *HCRTR1* rs2271933 genotype and in correlation with dimensional measures of agoraphobic cognitions in an extended sample of healthy probands. Finally, *HCRTR1* rs2271933 effects on behavioral avoidance and psychophysiological autonomic arousal during a behavioral avoidance task (BAT) were assessed in PD/AG patients. It was hypothesized that *HCRTR1* genotype-driven dysfunctional panic-related cognitions and behaviors as well as an oversensitivity of neural network and psychophysiological responses related to arousal and alerting might provide a pathomechanistic framework for an increased vulnerability to PD/AG as well as an impaired response to CBT.

## Materials and methods

For detailed information on samples, genotyping, CBT design, functional magnetic resonance imaging (fMRI) paradigm, BAT, and all statistical analyses refer to the Supplementary Methods.

### Samples

The discovery sample (‘MAC sample’) for the association study comprised a subsample of the two waves of the ‘Mechanism of Action in CBT’ study (MAC) funded by the German Federal Ministry of Education and Research (BMBF)^21^. It consisted of 483 participants of Caucasian origin with available blood samples and primary PD/AG (age = 35.1 ± 10.7 years, 341 females [70.6%], 24 cases without comorbid agoraphobia [5.0%], 127 cases with comorbid depression [26.3%], all cases free of psychotropic medication; for additional demographic information including PD/AG age of onset and therapeutic history see Supplementary Table 1). For categorical associations, an optimal 1:3 matching with healthy controls (HC) was performed (“optimal matching” as implemented in the R package MatchIt aims to minimize the global distance measure by matching samples with the smallest average absolute distance across all matched pairs, minimizing the distance between each pair^22,23^) based on age and sex against an available dataset of screened healthy controls recruited from the SFBTRR-58 subproject Z02 recruitment waves 1 and 2 (*n* = 2382; age = 25.2 ± 5.8 years, 1486 females [62.4%]). Matching was limited to a 1:3 ratio due to the availability of healthy female control samples (see below)^24^. In addition, the total sample of MAC PD/AG patients and SFBTRR-58 Z02 healthy controls had been characterized for scores on the German version of the Agoraphobic Cognitions Questionnaire (ACQ)^25^.

The replication sample (‘Münster sample’) consisted of 130 patients with primary PD/AG (age = 35.7 ± 11.1 years, 84 females [64.6%], 40 cases without comorbid agoraphobia [30.8%], 46 cases with comorbid depression [35.4%]) and 130 healthy controls (age = 35.8 ± 10.1 years) recruited at the Department of Psychiatry and Psychotherapy, University of Münster, Germany, supplemented with healthy controls recruited from the SFBTRR-58 subproject Z02 recruitment waves 1 and 2 and not used in the matching process for the discovery sample, in order to again reach a PD/AG:HC ratio of 1:3. Study inclusion criteria were comparable between the discovery and replication samples and based on DSM-IV criteria (see Supplementary Methods).

Patients and healthy controls gave full written informed consent; the studies were in agreement with the Declaration of Helsinki and approved by the respective local ethics committees.

### Cognitive-behavioral psychotherapy (CBT)

Within the MAC study, patients underwent CBT sessions following a manualized structure and administered twice weekly, with a total of 12 sessions^26^. A subsample of patients was available for genetic analyses (*n* = 189; age = 35.4 ± 11.0 years, 139 females [73.5%]).

Primary outcome measures investigated in the MAC study were the Hamilton Anxiety Rating Scale (HAM-A), the Clinical Global Impressions Scale (CGI), the Panic and Agoraphobia Scale (PAS), the number of panic attacks in the week prior to assessment and the Mobility Inventory for Agoraphobia Avoidance “Alone” Scale (MI), all of which were assessed at baseline and post CBT^26^.

### Functional MRI

A total of 94 healthy subjects of Caucasian descent performed the fMRI task (age = 28.5 ± 8.9 years, 63 females [67.0%], 44 samples drawn from the SFBTRR-58 subproject Z02 recruitment waves 1 and 2 and 50 samples additionally recruited at the Department of Psychiatry, Psychosomatics, and Psychotherapy, University of Würzburg, Germany). Analyses focused on neural activity of the alerting network as evaluated via the Attentional Network Task (ANT) capturing alerting, orienting and executive functions. The paradigm and data acquisition have been published previously (see Supplementary Methods)^19^.

### Behavioral avoidance task (BAT)

A subsample of the ‘MAC sample’ during recruitment wave 1 underwent the behavioral avoidance task^27,28^. The protocol for the BAT comprised of an exposure to a dark, small and closed test chamber which patients would first sit in front of with the door open for 10 min (‘anticipation phase’) before being locked in for a maximum of 10 min (‘exposure phase’), followed by the ‘recovery phase’, again, in front of the open door for 8 min. Avoidance behavior and psychophysiological measures were ascertained in all phases (see Supplementary Methods; for additional pre-treatment heart rate and pre- and post-treatment subjective fear readouts refer to the Supplementary Results and Discussion). Patients were explicitly allowed to terminate the BAT protocol at any time. Dependent on their behavior during the task, patients were categorized into one of three groups: ‘passive avoidance’ (no attempt of exposure in the BAT chamber), ‘active avoidance’ (flight during the 10 min period of exposure) and ‘no avoidance’ (no attempt of escape during the exposure). After completion of each phase, patients were instructed to rate their subjective experience of fear on a visual analogue scale of 1 to 10. Mean heart rates during the three phases were recorded via a continuous electrocardiogram.

Full *HCRTR1* rs2271933 genotype and BAT data was available for 271 PD/AG patients pre-CBT (age = 36.1 ± 10.9 years, 202 females [74.5%]) and for 183 patients post-CBT, excluding those randomized to a wait-list-control group (*n* = 49), treatment drop-outs (*n* = 33) and those who failed to repeat the BAT after therapy (*n* = 6).

### Genotyping

All samples were genotyped for the *HCRTR1* rs2271933 using a PCR-restriction-fragment-length-polymorphism (RFLP) assay (see Supplementary Methods).

## Results

### Categorical diagnosis of panic disorder with and without comorbid agoraphobia

*HCRTR1* rs2271933 allele and genotype frequencies and full test statistics, *p*-values, odds ratios (OR) and 95% confidence intervals (CI) for the discovery sample (MAC study) and the replication sample (Münster sample) are given in Table 1.

**Table 1:** Association studies of *HCRTR1* rs2271933 and PD/AG

							CA test						*χ*²-test						*χ*²-test	
				CC	CT	TT	*χ*²	*p*-value	CC/CT	TT	OR	95%CI	*χ*²	*p*-value	C	T	OR	95%CI	*χ*²	*p*-value
MAC	Total		Patients	120	212	151	20.62	**5.6** × **10**^**−6**^	332	151	1.78	1.41–2.24	23.65	**1.2** × **10**^**-6**^	452	514	1.41	1.22–1.64	21.31	**3.9** × **10**^−**6**^
			Controls	452	702	295			1154	295					1606	1292				
	Females		Patients	84	149	108	22.32	**2.3** × **10**^**-6**^	233	108	2.02	1.53–2.66	24.50	**7.4** × **10**^**-7**^	317	365	1.53	1.29–1.83	22.99	**1.6** × **10**^**−**^^**6**^
			Controls	337	495	191			832	191					1169	877				
	Males		Patients	36	63	43	1.14	0.285	99	43	1.34	0.88–2.05	1.62	0.203	135	149	1.16	0.89–1.52	1.06	0.304
			Controls	115	207	104			322	104					437	415				
Münster	Total		Patients	36	66	28	10.89	**9.7** × **10**^**−4**^	102	28	1.91	1.14–3.20	5.53	**0.019**	138	122	1.61	1.21–2.15	10.51	**1.2** × **10**^**−3**^
			Controls	163	178	49			341	49					504	276				
	Females		Patients	21	42	21	16.83	**4.1** × **10**^**−5**^	63	21	3.32	1.72–6.38	12.59	**3.9** × **10**^**−4**^	84	84	2.07	1.45–2.96	15.76	**7.2** × **10**^**−5**^
			Controls	111	118	23			229	23					340	164				
	Males		Patients	15	24	7	0.01	0.905	39	7	0.77	0.31–1.92	0.11	0.739	54	38	1.03	0.64–1.66	0.00	1.000
			Controls	52	60	26			112	26					164	112				

*PD/AG* panic disorder with and without agoraphobia, *MAC* discovery sample, *Münster* replication sample, *CA* Cochran–Armitage test for trend, *OR*  odds ratio, *CI* confidence interval

Significant *p*-values highlighted in bold (*p*-value < 0.05)

In the MAC discovery sample, rs2271933 T allele loading was significantly associated with PD/AG in the Cochran–Armitage test and the allelic Pearson’s *χ*²-test. Furthermore, the genotype model Pearson’s *χ*²-test supported a recessive effect of the T allele in PD/AG (TT vs CC/CT). Stratification by sex revealed a predominantly female association effect.

The association between the rs2271933 T allele and PD/AG was successfully validated in the Münster replication sample via the Cochran–Armitage test and Pearson’s *χ*²-tests for the allelic and recessive genotype models. Again, the genetic association was predominately driven by the female subsample of the Münster sample.

**Fig. 1:**
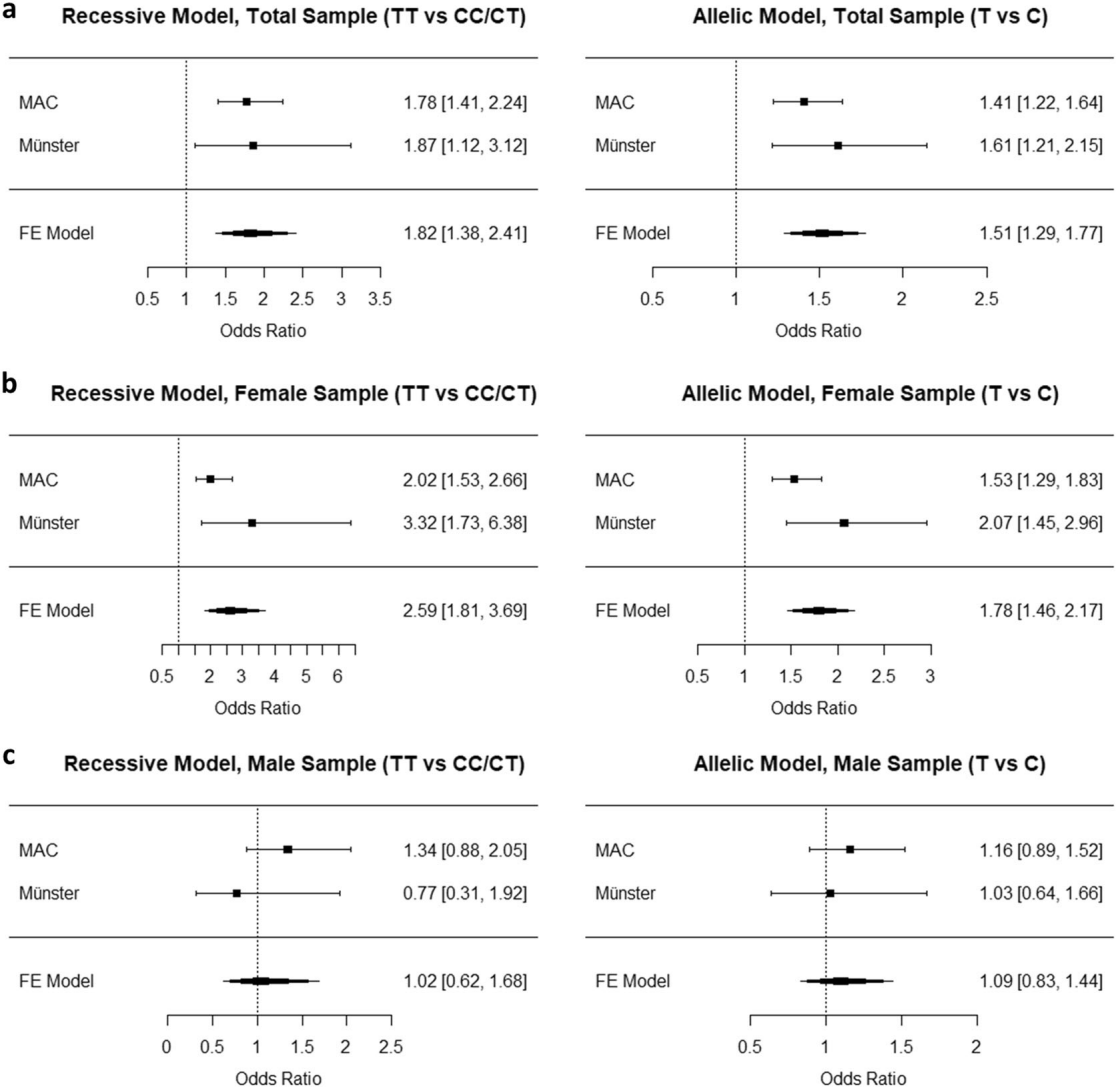
Forest plots of *HCRTR1* rs2271933 meta-analyses of association with PD/AG. PD/AG = panic disorder with and without agoraphobia; MAC = discovery sample; Münster = replication sample. FE = fixed-effects. Values in brackets represent 95% confidence intervals of odds ratios

Meta-analytical combination of the total discovery and replication samples (613 PD/AG patients, 1839 controls) in a fixed-effects model resulted in a significantly increased OR for both, the recessive (OR 1.82 [95%CI = 1.83–2.41], Z = 4.18, *p* = 2.9 × 10^−5^) and the allelic model (OR 1.51 [95%CI = 1.29–1.77], Z = 5.06, *p* = 4.2 × 10^−7^) (Fig. 1). Further meta-analyses confirmed the increased PD/AG OR in females only for the recessive (OR 2.59 [95%CI = 1.81–3.69], Z = 5.25, *p* = 2.0 × 10^−4^) and allelic model (OR 1.78 [95%CI = 1.46–2.17], Z = 5.73, *p* = 9.8 × 10^−9^).

### CBT treatment response in panic disorder with and without comorbid agoraphobia

In the MAC PD/AG patient subsample undergoing CBT (*HCRTR1* rs2271933 genotype distribution: CC = 58, CT = 78, TT = 53), TT genotype carriers displayed a significantly decreased treatment response as assessed via HAM-A scores (TT vs CC and TT vs CT), CGI scores (TT vs CC; trend towards significance for TT vs CT) and PAS scores (TT vs CC; trend towards significance for TT vs CT). Furthermore, in T allele homozygotes a trend towards a significantly diminished treatment response on the MI was discerned when compared to CT heterozygotes. Applying a recessive model (TT vs CC/CT) the reduced treatment response effects in T allele homozygotes remained significant for the HAM-A (*p* = 1.5 × 10^−3^), CGI (*p* = 0.031) and PAS (*p* = 0.044) scores. For full test statistics, *p*-values, ORs and 95% CIs refer to Table 2.

**Table 2:** Association studies of *HCRTR1* rs2271933 and CBT treatment response

			Baseline		Post CBT		Effect size				CT vs CC Effect size difference				TT vs CC Effect size difference				TT vs CT Effect size difference			
Outcome	Genotype	N	Mean	SD	Mean	SD	*d*	95%	CI	*p*-value	*d*	95%	CI	*p*-value	*d*	95%	CI	*p*-value	*d*	95%	CI	*p*-value
HAM-A^α^	CC	58	25.2	5.5	12.1	6.9	−2.5	−2.9	−2.1	4.83·10^−18^												
	CT	77	24.1	5.2	11.4	7.0	−2.6	−2.8	−2.3	8.33·10^−30^												
	TT	53	23.2	4.9	14.8	8.2	−1.7	−2.1	−1.3	1.56·10^−12^	−0.1	−0.5	0.4	0.764	0.6	0.1	1.1	**5.6·10** ^**−3**^	0.7	0.2	1.1	**1.3·10** ^**−3**^
CGI^α^	CC	58	5.2	0.7	3.2	1.1	−2.9	−3.3	−2.4	4.85·10^−18^												
	CT	77	5.2	0.8	3.4	1.0	−2.5	−2.9	−2.2	1.77·10^−22^												
	TT	53	5.3	0.7	3.8	1.1	−2.2	−2.6	−1.8	2.08·10^−14^	0.3	−0.3	0.8	0.150	0.7	0.1	1.3	**8.7·10** ^**−3**^	0.4	−0.1	1.0	0.062^+^
PAS^α^	CC	58	27.2	10.0	12.4	8.0	−1.5	−1.8	−1.3	5.92·10^−17^												
	CT	75	27.1	10.0	14.1	9.6	−1.3	−1.6	−1.1	4.33·10^−16^												
	TT	53	28.0	10.0	16.2	9.8	−1.2	−1.5	−0.9	8.59·10^−12^	0.1	−0.2	0.4	0.195	0.3	0.0	0.7	**0.017**	0.2	−0.1	0.5	0.077^+^
Panic attacks	CC	58	2.6	2.4	1.1	1.8	−0.4	−0.7	−0.2	1.84·10^−03^												
	CT	78	2.6	2.4	1.0	1.4	−0.5	−0.8	−0.3	3.17·10^−05^												
	TT	53	2.6	2.3	1.5	2.0	−0.3	−0.6	0.0	2.54·10^−02^	0.1	−0.1	0.3	0.129	0.1	−0.1	0.3	0.101	0.0	−0.1	0.2	0.396
MI	CC	49	2.9	0.9	1.9	0.8	−1.1	−1.4	−0.8	1.09·10^−10^												
	CT	66	2.9	0.7	1.8	0.7	−1.3	−1.5	−1.1	1.33·10^−18^												
	TT	53	3.0	0.9	2.0	0.9	−1.1	−1.4	−0.9	1.23·10^−12^	−0.2	−0.5	0.1	0.264	0.1	−0.2	0.4	0.347	0.2	−0.1	0.5	0.060^+^

Treatment effect size displayed as within- and between-groups Cohen’s *d* based on pre/post CBT score means and baseline standard deviation. Effect size differences based on robust linear regression were corrected for outcome’s baseline values

Significant effect size differences highlighted in bold (*p*-value < 0.05)

Note that the number of panic attacks in the previous week is also a sub-item of the integrated PAS score

*d* Cohen’s d effect size, *HAM-A* Hamilton Anxiety Rating Scale, *CGI* Clinical Global Impressions Scale, *PAS* Panic and Agoraphobia Scale, *MI* Mobility Inventory, *CI* confidence interval

^+^Trend^-^wise significance at *p*-value < 0.1

^α^ Significant effect size difference in a recessive design (TT vs CC/CT) with reduced treatment response in T allele homozygotes (for full statistical information see Supplementary Table 2)

### Dimensional anxiety traits in panic disorder with and without comorbid agoraphobia and in healthy controls

Within the total MAC PD/AG patient sample, T allele loading was significantly associated with increased ACQ scores (by 1.90 points per T allele, 95%CI: 0.76–3.04 points; allelic model, *p* = 0.027). When evaluating the effect of the rs2271933 T allele on ACQ scores within the sample of 2382 healthy subjects, T allele loading was again significantly associated with an increased ACQ score (by 0.50 points per T allele, 95%CI 0.35–0.65 points; allelic model, *p* = 0.014).

### Alerting network in healthy controls

**Fig. 2:**
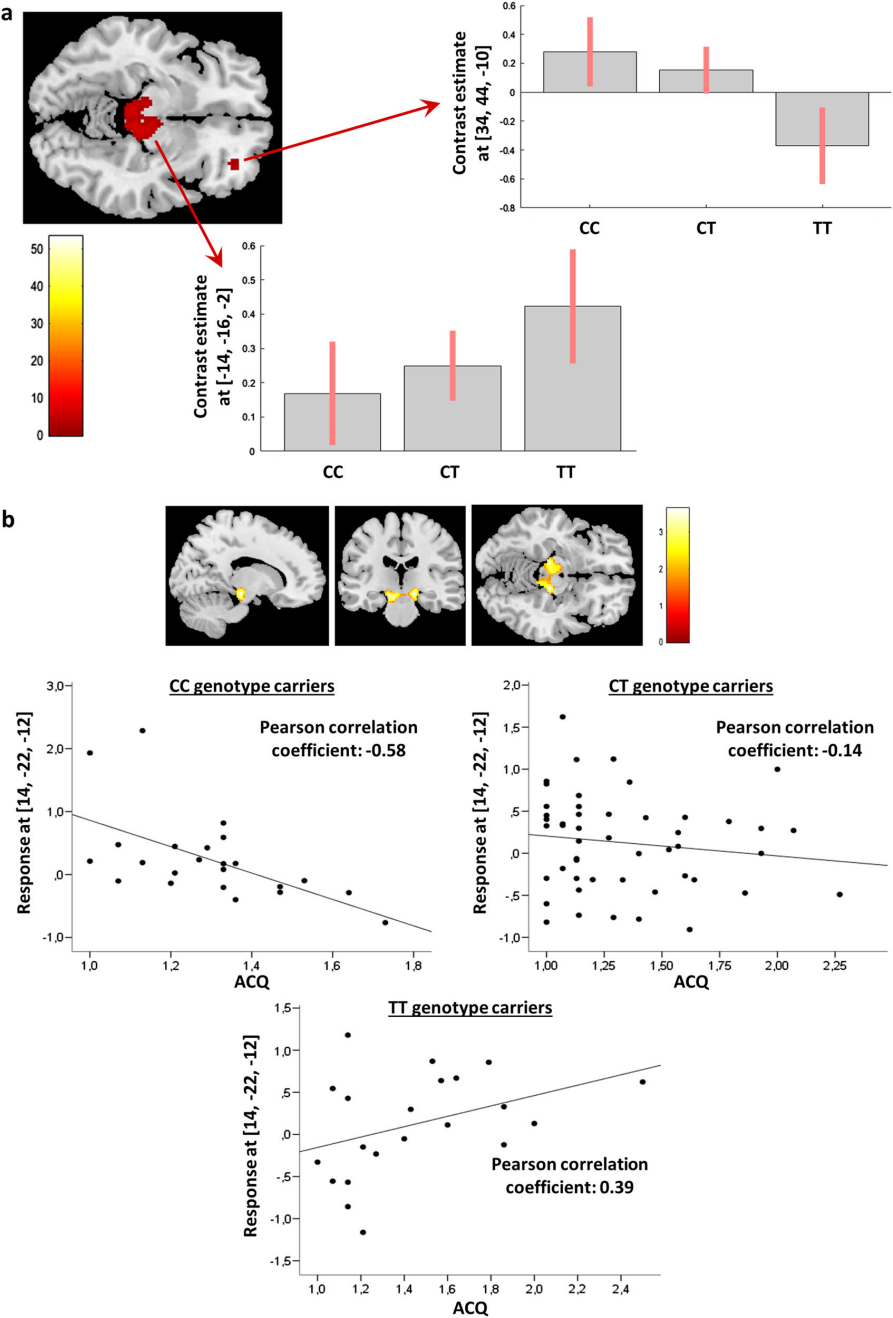
*HCRTR1* rs2271933 genotypes and the alerting system. **a** Left: Significant *HCRTR1* rs2271933 gene-dose effect clusters in the alerting system are overlaid on a single subject T1 anatomical image. Right and bottom: Bar plots represent the *HCRTR1* rs2271933 genotype-dose effect in terms of contrast estimates at the local maxima. **b** Effects of Agoraphobic Cognitions Questionnaire (ACQ) scores on the alerting network. Top: Activation clusters rendered on a single subject brain surface. Bottom: Correlations of ACQ scores with locus coeruleus (LC) activation. In *HCRTR1* rs2271933 T allele homozygotes, ACQ scores correlated positively with LC neural activity, while there was a negative correlation in C allele homozygotes (see Results)

In the sample of healthy probands performing the fMRI Attention Network Task (*HCRTR1* rs2271933 genotype distribution: CC = 23, CT = 50, TT = 21), two significant clusters of neural activation in the alerting condition were detected when applying a gene dosage analysis (Fig. 2a): One cluster within the right inferior frontal gyrus (IFG, *x* = 34, *y* = 44, *z* = −10) with decreasing activation associated with increased T allele counts (*Z* = 3.1, *p* = 0.041, FDR-corrected at the voxel level) and one cluster within the bilateral locus coeruleus (LC, *x* = −14, *y* = −16, *z* = −2) with increasing activation associated with increased T allele counts (*Z* = 3.2, *p* = 0.031, FDR-corrected at the voxel level).

In order to address the relation between neural activation of the alerting system, rs2271933 T allele count and dimensional anxiety traits (ACQ), interaction analyses were performed. Neural activation in the LC cluster significantly correlated with increased ACQ scores in TT genotype carriers and decreased ACQ scores in CC genotype carriers (Fig. 2b) (TT > CC/CT: *Z* = 3.5, *p* = 0.032; TT > CC: *Z* = 3.6, *p* = 0.028; TT > CT: *Z* = 1.5, *p* = 0.210). A negative correlation between ACQ scores and right IFG activation in TT genotype carriers reached trend-wise significance (TT < CC/CT: *Z* = 2.1, *p* = 0.084; TT < CC: *Z* = 1.9, *p* = 0.089; TT < CT: *Z* = 0.01, *p* = 0.981).

There was no genotype effect on accuracy and reaction time on the behavioral level. To assess the relation between ACQ scores and behavioral parameters, partial correlations were performed using sex as nuisance variable: ACQ scores did not significantly influence behavioral performance (*R*_accuracy_ = 0.039, *p* = 0.714; *R*_reaction time_ = −0.055, *p* = 0.600, *R*_alerting_ = 0.075, *p* = 0.480).

### Behavioral avoidance task (BAT) in panic disorder with and without comorbid agoraphobia

**Fig. 3:**
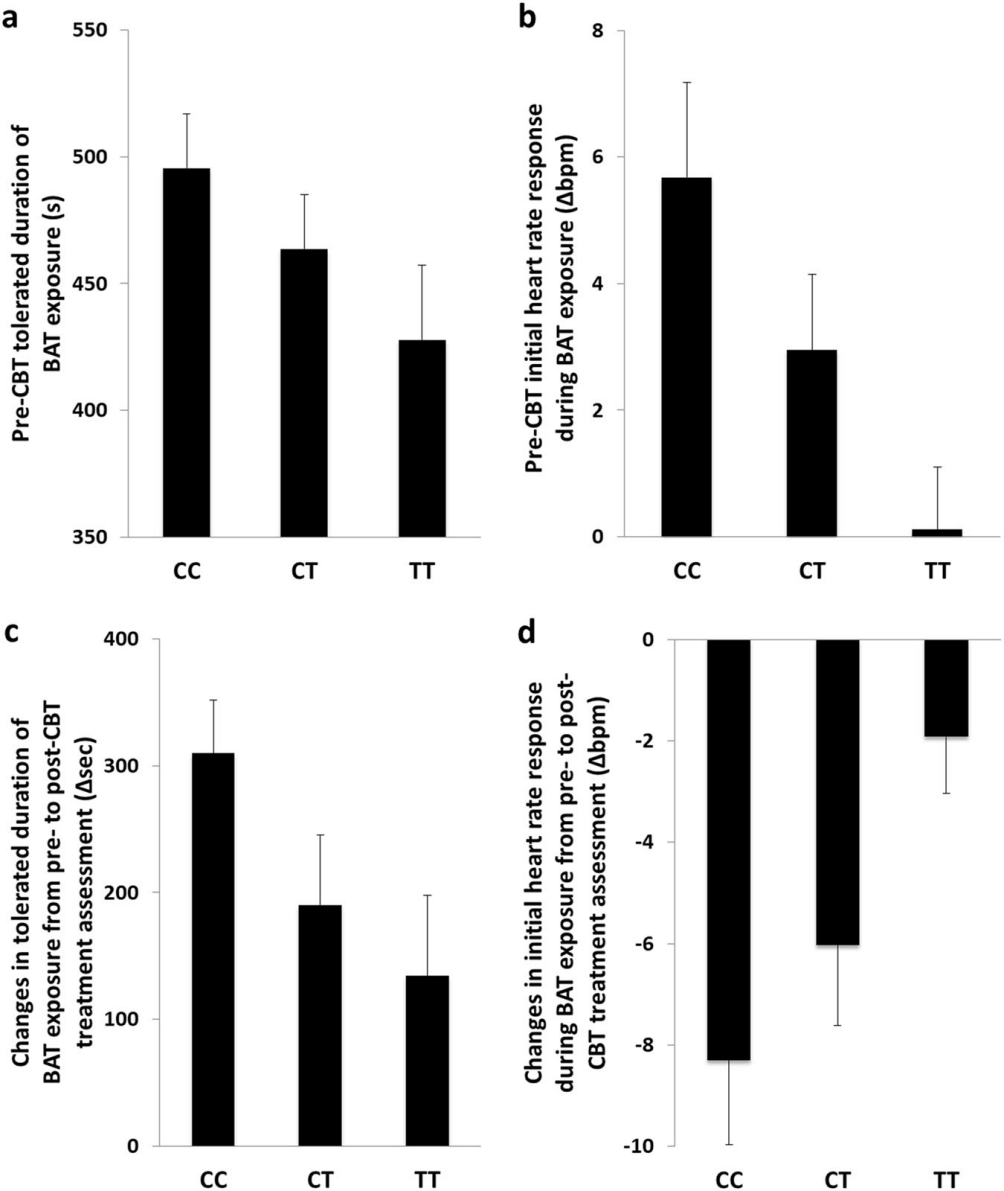
*HCRTR1* rs2271933 T allele loading and behavioral avoidance task (BAT) outcomes. **a** T allele loading vs pre-treatment (pre-CBT) duration of exposure (s). **b** T allele loading vs pre-treatment initial heart rate response (∆bpm) during exposure, i.e., increase from last minute of anticipation to first minute of exposure, in non-avoiding patients. **c** T allele loading vs the pre- to post-treatment difference in tolerated duration of exposure (∆s). **d** T allele loading vs the pre- to post-treatment difference of the initial heart rate response (∆bpm) during exposure. All values are presented as means ± standard error (SE)

*Pre-treatment assessment* (*HCRTR1* rs2271933 genotype distribution: CC = 79, CT = 120, TT = 72): During the BAT, frequency of avoidance behavior was significantly more pronounced along with an increasing number of rs2271933 T alleles (categorical BAT analysis; passive avoidance by genotype: CC = 2 [2.5%], CT = 15 [12.5%], TT = 12 [16.7%]; active avoidance by genotype: CC = 19 [24.1%], CT = 21 [17.5%], TT = 15 [20.8%]; no avoidance by genotype: CC = 58 [73.4%], CT = 84 [70.0%], TT = 45 [62.5%]; linear-to-linear trend: *χ*²(1) = 5.172, *p* = 0.023). Concordantly, mean duration of tolerated BAT exposure trended towards a significant decrease with increasing T allele loading (dimensional BAT analysis; linear trend *p* = 0.073; Fig. 3a).

In those patients entering the test chamber, the mean heart rate during BAT anticipation did not significantly differ between patients showing active avoidance vs no avoidance during BAT exposure (Behavior F(1,186) = 0.338, *p* = 0.562). Also, no genotype effect was observed during the anticipation phase (Genotype F(2,186) = 1.718, *p* = 0.182; Genotype × Behavior F(2,186) = 2.476, *p* = 0.087). However, while the initial heart rate increase during BAT exposure did not differ between genotype groups in active avoiders (F(2,42) = 0.615, *p* = 0.546), the heart rate response decreased with increasing number of T alleles in non-avoiding patients (linear trend: *p* = 7.7·10^−3^; Fig. 3b).

*Post-treatment assessment* (*HCRTR1* rs2271933 genotype distribution: CC = 57, CT = 75, TT = 51): In patients showing active or passive avoidance behavior during the BAT assessment prior to therapy, the increase of the mean duration of tolerated BAT exposure from pre- to post-assessment diminished with an increasing number of rs2271933 T alleles (linear trend *p* = 0.043; Fig. 3c).

Accordingly, the pre- to post-assessment reduction of the initial heart rate response from anticipation to exposure phase attenuated with an increasing number of rs2271933 T alleles (linear trend *p* = 6.0 × 10^−3^; Fig. 3d) irrespective of whether patients showed active avoidance or not during the BAT pre-treatment assessment (Genotype × Behavior F(2,119) = 0.294, *p* = 0.746).

## Discussion

Our data demonstrate an association between the *HCRTR1* rs2271933 T allele and PD/AG in a Caucasian population, including a successful replication and meta-analysis. The *HCRTR1* rs2271933 driven non-synonymous Ile408Val substitution resides in the receptor’s C-terminus and is therefore likely to be part of a domain involved in protein-protein interaction, but as of yet, it remains to be experimentally tested, whether the SNP affects expression levels, effector coupling or dimerization of the OX_1_ receptor^29^. Based on the above mentioned findings the T allele — presently discerned to mediate hyperarousal- and/or panic-related anxiety behavior — is hypothesized to enhance OX_1_ receptor signaling (e.g., on the expression level, or due to reduced cycling/desensitization) and thereby to increase the orexinergic tone in key brain areas of the arousal/alerting system. Interestingly, the genetic association was mainly driven by the female subsamples of the present samples, a phenomenon already observed in other neuropeptide- and monoamine/catecholamine-related polymorphisms implicated in PD/AG pathology^30,31^. Indeed, sexually dimorphic expression of orexin and the orexin receptors in the CNS has been described in mice^32^. Furthermore, it has been shown that restraint stress-induced HPA-axis activation and cognitive impairments via increased glucocorticoid receptor-dependent orexin expression occur exclusively in female rats and can be prevented by chemogenetic inhibition of hypothalamic orexinergic neurons, pointing to a sex-specific role of orexins and their receptors in stress susceptibility^33^. The only other study evaluating orexin receptor polymorphisms in PD/AG failed to show genetic association for *HCRTR1* rs2271933, but rather reported a significant finding for the *HCRTR2* rs2653349 A allele, again predominantly in the female subsample^15^. It should be noted, however, that in this previous study PD/AG sample size was limited, controls were not screened for PD/AG and no independent replication was provided.

Moreover, the *HCRTR1* rs2271933 T allele was found to confer significantly poorer outcomes on clinical assessments (HAM-A, CGI) and a self-report measurement (PAS) following exposure-based CBT. Given that changes in vigilance for threat information have been demonstrated to precede and predict clinical changes in exposure-based CBT for PD/AG^34^, an altered orexinergic tonus differentially driven by rs2271933 genotype and affecting arousal/alerting processes might influence CBT outcome in PD/AG. The present finding extends the emerging body of evidence for biomarkers of psychotherapy response prediction in PD/AG, so far mainly focusing on genetic variations related to serotonergic and monoaminergic neurotransmission such as the monoamine oxidase A (*MAOA*) upstream variable number of tandem repeats (uVNTR) SNP^35^, to the arousal-related orexin system.

In the attempt to explore intermediate phenotypic levels potentially contributing to the observed genotype effects, the *HCRTR1* rs2271933 T allele count was found to be associated with increased agoraphobic cognitions (ACQ) scores within the MAC PD/AG patient sample and in a large sample of psychiatrically healthy subjects. It has already been shown that increased ACQ scores correlate directly with increased bodily sensations in feared situations in anxiety disorder patients and in psychiatrically healthy controls^36^. Accordingly, elevated ACQ scores prior to a CO_2_ challenge predicted the intensity of frightening cognitions after inhalation, both in a PD/AG and a healthy control sample^37^, thus providing a cognitive link between arousal-related interoceptive sensations and PD/AG risk. Also, the presently observed therapygenetic effect might in part be explained by the fact that the *HCRTR1* rs2271933 T allele predicted increased ACQ scores, given that catastrophic misinterpretations during a 12-week CBT program have previously been shown to impair therapeutic success^38^. In sum, the *HCRTR1* rs2271933 T allele might confer an increased proneness to PD/AG-related catastrophizing thought patterns and thereby increased PD/AG risk as well as treatment failure.

On a neural level, we observed a significant effect of *HCRTR1* rs2271933 genotype on alerting network activation in healthy subjects, with an increased T allele count associated with decreased neural response in the right inferior frontal gyrus (IFG) and an increased response in the locus coeruleus (LC). Remarkably, LC activation varied positively in function of the ACQ in T allele homozygotes, signifying that a higher ACQ score was linked to a stronger neural bottom-up processing of internal stimuli. At the same time, reduced activation in the IFG, a region which has been hypothesized to represent the modulation of warning signals on the level of alertness, might indicate an impaired top-down inhibition of the subcortical alerting network^39^. Orexin has been reported to activate noradrenergic LC neurons and thereby heightens arousal^40^. Notably, in terms of fear conditioning *Hcrtr1* knockout mice displayed impaired freezing responses and reduced neural activation of the lateral amygdala, which could be rescued by adeno-associated viral-mediated restoration of *Hcrtr1* expression in the LC^8^. Local microinjections of an OX_1_ receptor antagonist and subsequent optogenetic specific stimulation of orexin fibers in the LC further validated the importance of OX_1_ receptor-controlled norepinephrinergic LC neurons in the formation of fear memories^9^. Additionally, the information that hypercapnia leads to increased brainstem activation in PD/AG patients compared to healthy controls^41^, and the positive correlation between LC activation and ACQ scores in TT genotype carriers, advocate for an increased risk of developing PD/AG due to a combination of elevated attention to autonomic bodily functions and increased somatic arousal due to dysfunctional cortical attentional networks^20,42^. Moreover, overactivation of the LC-amygdala circuit has been linked to increased fear generalization, which was reversible under OX_1_ receptor antagonist treatment^43^. Since individuals with higher trait anxiety and amygdala hyperresponsivity have been proven to exhibit an increased resistance to fear extinction^44^, overactivation of the LC in alerting conditions presently identified in *HCRTR1* rs2271933 T allele homozygotes, may have significantly impaired CBT efficiency of exposure-based extinction learning, as observed in the treatment response analysis. In sum, the present results propose a *HCRTR1* genotype by phenotype interaction in terms of an imbalance between frontal top-down and brain stem bottom-up control in the alerting network, reflecting a putative neuronal model of hyperarousal in the perception and processing of fear-related bodily symptoms with relevance to PD/AG pathology as well as therapy resistance due to abnormal stimulus generalization and extinction.

Finally, the *HCRTR1* rs2271933 T allele was associated with increased rates of avoidance behavior and a decreased capacity to endure psychophysiological activation in PD/AG patients during a fear-provoking behavioral avoidance test (BAT). Surprisingly, T allele loading was associated with decreased physiological fear reactivity to BAT exposure as indicated by the measured heart rate responses, however, only in BAT non-avoiding but not in BAT escaping patients. In T allele homozygotes displaying no active avoidance, no substantial fear increase from anticipation to exposure was observed. Thus, if the patients’ arousal system is activated, T allele carriers might be significantly more likely to avoid the fear-inducing situation, manifesting in escape from the phobic environment, resulting in more frequent avoidance behavior and, in turn, lower levels of autonomic fear in non-avoiding subjects as compared to non-risk-allele carriers. Our results are in line with current learning theories in PD/AG, stating that chances of experiencing another panic attack are heightened once they have been associated with internal cues of elevated arousal. This could explain why T allele carriers would most likely choose to avoid phobic exposure in general and thus are more prone to develop PD/AG from an initial panic attack^45^. Additionally, we found an increased maintenance of avoidance behavior during the BAT after CBT treatment with an increasing number of rs2271933 T alleles that went along with a lack of a reduction of physiological fear reactivity from pre- to post-treatment assessment. Reduced tolerance of fear activation during phobic exposure — as displayed by T allele carriers — could possibly be detrimental for safety learning and thus impede successful fear extinction, as observed in the treatment response analysis^46^. In line with this hypothesis, agoraphobic avoidance was the most consistent predictor of decreased improvement in CBT for PD/AG in a recent systematic literature review^47^. Taken together, the present data support the notion, that the *HCRTR1* rs2271933 T allele might confer a genetic risk of decreased resilience to phobic exposure in PD/AG resulting in pronounced avoidance behavior.

The present results should be considered in the light of some limitations. Besides the necessity to replicate our findings in even larger samples, particularly when stratifying for sex, several factors not accounted for and thus potentially confounding the present results such as possible moderation effects by childhood trauma and recent life events should be controlled for in future studies. Given that the healthy controls were younger than the PD/AG patient populations, despite optimal matching of the available samples, we cannot exclude a potential bias in the results given the possibility that they might develop a PD/AG later in life. Along these lines, epigenetic mechanisms like DNA methylation mediating environmental influences and governing gene function remain to be addressed^48^. The decision to investigate only one SNP in the *HCRTR1* gene was based on its likely functional relevance and due to it having been investigated in the majority of association studies on *HCRTR1* gene variation in neuropsychiatric phenotypes^15–18^. However, in order to capture the entire *HCRTR1* gene information and to exclude potential effects of population stratification, a tagging SNP approach would have to be applied. Additionally, it should be noted that SNPs in linkage disequilibrium with rs2271933 could causally drive the reported findings, yet this would optimally require whole genome sequencing data to be ruled out entirely. Future studies are warranted to experimentally delineate the presently only assumed functionality of *HCRTR1* rs2271933 and its interaction with other established psychiatric candidate gene variants. Particularly, the interaction of *HCRTR1* and *HCRTR2* gene variation remains to be elucidated given preclinical evidence for a bi-directional control of anxiety via orexin receptors^49^.

In conclusion, we have gathered converging evidence for a both patho-genetic and therapy-genetic role of the *HCRTR1* rs2271933 T allele in PD/AG. In synopsis with translational preclinical studies, the present findings suggest a potential value of OX_1_ receptor antagonists as a novel treatment strategy in arousal- and panic-related anxiety^10–13^. Also, applying a personalized treatment approach, *HCRTR1* gene variation, along with physiological, neurocircuit, behavioral and self-report measures, might aid in predicting treatment response and allow for tailoring therapeutic interventions to the individual patient’s risk factor constellation^50,51^.

## Supplementary information

Supplementary File
