# Supplementary material for: Orexin in the anxiety spectrum: association of a HCRTR1 polymorphism with panic disorder/agoraphobia, CBT treatment response and fear-related intermediate phenotypes
Source: Transl Psychiatry. 2019 Feb 4;9:75. doi: 10.1038/s41398-019-0415-8 (PMC6361931; doi:10.1038/s41398-019-0415-8)
Supplement: Supplementary file 1 — Supplementary File [file 41398_2019_415_MOESM1_ESM.docx]

**Supplementary Methods**

## **Samples**

Discovery sample: In the MAC study, diagnosis of PD/AG was established via structural application of the Composite International Diagnostic Interview (CIDI) according to DSM-IV criteria[^1^](#_ENREF_1). All patients were free of psychotropic medication. In the SFBTRR-58 subproject Z02 recruitment waves 1 and 2, trained psychologists established the absence of manifest or lifetime mental axis one disorders via the Mini International Neuropsychiatric Interview following DSM-IV criteria[^2^](#_ENREF_2). Inclusion criteria included: Caucasian background (up to third generation; based on self-report), right-handedness, and fluency in German. Exclusion criteria included: Pregnancy, severe medical conditions, intake of centrally active medication, and illegal drug abuse. For more information on the SFBTRR-58 subproject Z02 recruitment waves 1 and 2 refer to Klauke *et al.*[^3^](#_ENREF_3) and Schiele *et al.*[^4^](#_ENREF_4).

Replication sample: In the Münster study, the diagnosis of PD/AG was ascertained by experienced psychiatrists on the basis of medical records and structured clinical interviews according to the criteria of DSM-IV[^5^](#_ENREF_5).

Patients with comorbid psychotic disorders, bipolar disorder, disorders due to psychoactive substance use, current suicidal intent, borderline personality disorder, any form of mental retardation, known neurological, neurodegenerative or relevant somatic disorders were excluded in both, the discovery and the replication sample. Patients receiving psychotherapeutic or psychopharmacological treatment for PD/AG or another mental disorder, or had contraindications of exposure-based CBT were excluded from the MAC study.

## **Genotyping**

Standard de-salting procedures were used to isolate genomic DNA samples from venous EDTA-blood. Samples were genotyped for *HCRTR1* rs2271933 according to the following PCR-restriction fragment length polymorphism (RFLP) procedure: DNA was amplified by PCR with the oligonucleotide primers Forward: 5´-CCAGAGTCACACAGGCAGAAA-3´ and Reverse: 5´-CCTGCTGCATCTGTCTCCTTAT-3´. PCR products were incubated with the restriction endonuclease BsmBI (NEB, Frankfurt a. Main, Germany) at 55°C for 2 hours resulting in differentially sized fragments (C = 359 bp, T = 195 bp + 164 bp). Fragments were separated on a 4% agarose gel by electrophoresis and visualized with ethidium bromide. Fragment lengths and resulting genotypes were determined by two independent investigators blinded for diagnosis.

## **Genetic association**

Hardy-Weinberg equilibrium was evaluated based on χ² statistic with 10,000 permutations applying the R package ‘HardyWeinberg’[^6^](#_ENREF_6) and did not significantly differ from population estimates in the discovery sample or the replication sample (*p*=0.28 and 0.27, respectively). *HCRTR1* rs2271933 allele and genotype distributions were analyzed by Cochran–Armitage tests for trend (TT>CT>CC) and Pearson’s χ² test of independence (T vs C). Additionally, a grouped recessive genotype model (TT vs CC/CT) was tested via Pearson’s χ² tests. Odds ratios (ORs) and 95% confidence intervals (CIs) were calculated based on contingency tables and logistic regression. Meta-analytical OR combinations of the MAC and Münster samples were calculated based on fixed-effects models, separately for the allelic and the genotype design. All analysis steps were run in the total sample and in the female and male subsamples, respectively. Linear regression models were used to evaluate the relationship of allele and genotype distributions and Agoraphobic Cognitions Questionnaire (ACQ)[^7^](#_ENREF_7) scores. In each case an ‘allele’ (T allele count 0 vs 1 vs 2) and a ‘genotype’ (CC vs CT vs TT) linear model were fitted with the intermediate phenotype score as the outcome. In absence of a significant difference (*p*-value>0.05) the more parsimonious allelic model was chosen over the genotype model. Calculations were performed in R version 3.3 using the packages MatchIt, coin, fsmb and metafor.

## **Treatment response analysis**

Full clinical follow-up data and *HCRTR1* rs2271933 genotypes were available for 189 PD/AG patients of the first wave of the multicenter psychotherapy study (MAC study; see above) evaluating exposure-based CBT[^8^](#_ENREF_8).

Primary outcome measures in the treatment response analyses were the Hamilton Anxiety Rating Scale (HAM-A)[^9^](#_ENREF_9) score, the Clinical Global Impression Scale (CGI)[^10^](#_ENREF_10) score, the Panic and Agoraphobia Scale (PAS)[^11^](#_ENREF_11) score, the number of panic attacks in the week prior to assessment (part of the PAS) and the Mobility Inventory for Agoraphobia Avoidance “Alone” Scale (MI)[^12^](#_ENREF_12) score. All analyses were performed with quantitative outcome variables.

Effect size differences comparing baseline and post-CBT treatment values of each primary outcome measure were standardized with the pooled standard deviation at baseline and adjusted for baseline values to account for regression to the mean. For each outcome, 1) an ordinary least squares linear regression, 2) a linear regression with robust standard errors and 3) a robust linear regression were fitted. Since the statistical outcome differed between the models, results of the robust linear regression (3) were reported, which make the fewest model assumptions. We used one-sided Wald χ² tests for the grouped genotype association (CT vs CC, TT vs CC, TT vs CT; T allele = risk allele).

## **Functional MRI of the alerting network**

The Edinburgh Handedness Inventory was used to ascertain right-handedness[^13^](#_ENREF_13).

The attention network task (ANT) represents a combination of the cued reaction time[^14^](#_ENREF_14) and flanker task[^15^](#_ENREF_15) and defines the alerting condition as achieving and maintaining an alerting state, with the alerting effect calculated by subtracting the double cue conditions (information that the target stimulus will appear) from the no cue conditions (no information that the target stimulus will appear)[^14^](#_ENREF_14)^,^ [^16^](#_ENREF_16)^,^ [^17^](#_ENREF_17). During the behavioral analysis mean reaction times and accuracy were calculated for all participants.

Functional imaging data was acquired in a 3T TIM Trio Scanner (Siemens, Erlangen, Germany) with a gradient-echo EPI sequence (repetition time (TR)=2000 ms, echo time (TE)=30 ms, field of view (FoV)=192 mm, 3 mm thickness, 36 slices, 420 volumes).

Data processing was performed using Statistical Parametric Mapping version 12 (SPM12, <http://www.fil.ion.ucl.ac.uk/spm/>). Preprocessing included steps of temporal and spatial alignment (slice time correction and realignment & unwarp), spatial normalization into a standard stereotactic space (MNI, Montreal Neurological Institute, Quebec, Canada) and spatial smoothing with an isotropic Gaussian kernel of 8 mm at full width at half maximum (FWHM). On single subject level, the general linear model (GLM) approach was used. The alerting contrast was defined as ‘no cue’ minus ‘double cue’ condition as suggested by Fan *et al.* (2005)[^17^](#_ENREF_17). In addition, explicit masking was applied using a network mask including the right middle and superior frontal gyri (rMFG, rSFG), the right superior and inferior parietal lobules (rSPL, rIPL) and the brainstem including the locus coeruleus (LC). The mask was defined using the Wake Forest University PICKATLAS (www.fmri.wfubmc.edu) as implemented in SPM.

First, it was addressed how the genotype influenced neural processing of the alerting network, in terms of which brain activations were activated when an alerting cue was presented (double cue) in contrast to when there was no cue. Potential gene-dosage effects were identified in the alerting network using ANOVA models with the T allele count (0 vs 1 vs 2) as the independent factor, alerting activation maps as the dependent factor as well as sex as nuisance variable. Subsequently, the correlation between ACQ scores and neural activation was analyzed: individual psychometric ACQ scores entered the one-way ANCOVA as covariate of interest and post hoc T-tests followed up on the genotype-specific influence of psychometric score and neural activation (genotype $\times$ ACQ interaction). Multiple-hypothesis corrections were conducted according to the Benjamini-Hochberg procedure[^18^](#_ENREF_18), with adjusted levels of *p*-value<0.05 at the voxel-level considered significant.

## **Psychophysiological assessment in a behavioral avoidance task (BAT)**

Due to missing data and the exclusion of those patients taking beta-blockers heart rate data were available in 192 patients (*HCRTR1* rs2271933 genotype distribution: CC=54, CT=86, TT=52) and 125 patients (CC=37, CT=51, TT=37) during pre- and post-CBT BAT assessment, respectively.

To test for possible effects of genotype on avoidance behavior, a χ² test for linear trend was conducted with the number of rs2271933 T alleles (0 vs 1 vs 2) and behavior (passive avoidance vs active avoidance vs no avoidance) as between-subject factors. In addition, a model of variance was applied including genotype as a between-subject factor to test for genotype effects on tolerated duration of BAT exposure. To test the effect of genotype on reported fear and heart rate, respectively, we applied a mixed-model analysis of variance including genotype as a between-subject factor and BAT phases (anticipation vs exposure vs recovery) and in case of pre-to-post assessment analyses time as a within-subject factor in those patients entering the test chamber (excluding passive avoiders with no available data during exposure phase). To control for presently observed effects of active avoidance on reported fear and heart rate in the mixed-model analysis of variance, behavior (active avoidance vs no avoidance) was also included as a between-subject factor. In the case of heart rate, we additionally tested for genotype effects on the initial response during BAT exposure (increase from last minute of anticipation to first minute of exposure) because of its high sensitivity for fear processing as demonstrated previously[^19^](#_ENREF_19). For the results and discussion of the subjective fear analysis refer to the Supplementary Results and Discussion.

**Supplementary Results**

**Behavioral Avoidance Task (BAT) – Heart rate**

*Pre-treatment assessment*: During the recovery phase, the heart rate did not significantly differ between genotype groups (Genotype F(2,189)=0.432, *p*=0.650), indicating comparable baseline levels during resting state. During the anticipatory phase, heart rate measures were significantly higher as compared to the recovery phase (Phase F(1,186)=16.110, *p*<0.001; anticipatory phase mean HR=76.94 bpm, SD=10.81 vs. recovery phase mean HR=75.49 bpm, SD=9.22). This effect, however, was not modulated by genotype (Phase $\times$ Genotype F(2,186)=0.903, *p*=0.407; Phase $\times$ Genotype $\times$ Behavior F(2,186)=0.121, *p*=0.886). Taken together, we observed no genotype effect on heart rate levels during the BAT phases of anticipation or recovery.

**Behavioral Avoidance Task (BAT) – Subjective Fear Readouts**

*Pre-treatment assessment*: In contrast to the autonomic arousal, a higher reported fear during the BAT anticipation phase significantly predicted active avoidance during BAT exposure (Behavior F(1,236)=16.057, *p*=8.2·10^-5^) irrespective of *HCRTR1* rs2271933 genotype group (Genotype $\times$ Behavior F(2,236)=2.150, *p*=0.119). However, overall levels of reported fear during BAT anticipation significantly differed between genotype groups (Genotype F(2,236)=3.462, *p*=0.033) with the highest level in T alleles homozygotes (TT=3.5±0.3 points; CC=3.2±0.2; CT=2.9±0.2). Again contrary to the heart rate data no genotype effect on the increase of reported fear from anticipation to BAT exposure was observed (Genotype F(2,236)=1.399, *p*=0.249) in both active-avoiding and non-avoiding patients (Genotype x Behavior F(2,236)=0.856, *p*=0.426).

*Post-treatment assessment*: Genotype did not affect the decrease of reported fear from pre to post BAT assessment.

**Supplementary Discussion**

In addition to the reported effects on avoidance behavior and heart rate response, the present results are further supported by the subjective fear outcome analysis. As compared to C allele carriers, T allele homozygotes reported higher anticipatory anxiety that, importantly, was demonstrated to predict avoidance behavior during BAT exposure. A pronounced threat expectancy (especially in TT genotype carriers) might trigger acute fear responses and avoidance behavior. T allele carriers would be more likely to be affected by PD/AG after the occurrence of their first panic attack and would also be more prone to escape or avoid fear-relevant exposure in accordance with current PD/AG disease concepts. Specifically, a heightened incidence rate of panic attacks would follow the association of increased arousal with subjective internal cues and their subsequent misinterpretation as threats, spiraling into even more maladaptive attention to interoception[^20^](#_ENREF_20)^,^ [^21^](#_ENREF_21).

**Supplementary Tables**

**Supplementary Table 1**: Patient samples demographic overview.

Legend to supplementary table 1: The ”medication” column refers to any form of psychopharmacological treatment at the time of blood sampling. PD/AG = panic disorder with and without agoraphobia; MAC = discovery sample; Münster = replication sample; NA = information not available.

**Supplementary Table 2**: Association studies of *HCRTR1* rs2271933 and CBT treatment response in a recessive model (TT vs CC/CT).

Legend to table 2: Treatment effect size displayed as within- and between-groups Cohen’s *d* based on pre/post CBT score means and baseline standard deviation. Effect size differences based on robust linear regression were corrected for outcome’s baseline values. Significant effect size differences highlighted in bold (*p*-value<0.05). *d* = Cohen’s d effect size; HAM-A = Hamilton Anxiety Rating Scale; CGI = Clinical Global Impressions Scale; PAS = Panic and Agoraphobia Scale; MI = Mobility Inventory; CI = confidence interval. Note that the number of panic attacks in the previous week is also a sub-item of the integrated PAS score.

## **References**

1. Wittchen HU, Garczynski E, Pfister H. Composite International Diagnostic Interview According to ICD-10 and DSM-IV. Göttingen, Germany Hogrefe; 1997.

2. Sheehan DV, Lecrubier Y, Sheehan KH, Amorim P, Janavs J, Weiller E, et al. The Mini-International Neuropsychiatric Interview (M.I.N.I.): the development and validation of a structured diagnostic psychiatric interview for DSM-IV and ICD-10. J Clin Psychiatry 1998; **59 Suppl 20**: 22-33;quiz 4-57.

3. Klauke B, Deckert J, Reif A, Pauli P, Zwanzger P, Baumann C, et al. Serotonin transporter gene and childhood trauma--a G x E effect on anxiety sensitivity. Depress Anxiety 2011; **28**: 1048-57.

4. Schiele MA, Ziegler C, Holitschke K, Schartner C, Schmidt B, Weber H, et al. Influence of 5-HTT variation, childhood trauma and self-efficacy on anxiety traits: a gene-environment-coping interaction study. J Neural Transm (Vienna) 2016; **123**: 895-904.

5. Wittchen HU, Zaudig M, Fydrich T. SKID-I: strukturiertes klinisches Interview für DSM-IV, Achse I : Psychische Störungen. Göttingen, Germany: Hogrefe; 1997.

6. Graffelman J. Exploring Diallelic Genetic Markers: The HardyWeinberg Package. Journal of Statistical Software 2015; **64**: 1-23.

7. Chambless DL, Caputo GC, Bright P, Gallagher R. Assessment of fear of fear in agoraphobics: the body sensations questionnaire and the agoraphobic cognitions questionnaire. J Consult Clin Psychol 1984; **52**: 1090-7.

8. Gloster AT, Wittchen HU, Einsle F, Lang T, Helbig-Lang S, Fydrich T, et al. Psychological treatment for panic disorder with agoraphobia: a randomized controlled trial to examine the role of therapist-guided exposure in situ in CBT. J Consult Clin Psychol 2011; **79**: 406-20.

9. Shear MK, Vander Bilt J, Rucci P, Endicott J, Lydiard B, Otto MW, et al. Reliability and validity of a structured interview guide for the Hamilton Anxiety Rating Scale (SIGH-A). Depress Anxiety 2001; **13**: 166-78.

10. Guy W. ECDEU assessment manual for psychopharmacology. Rockville, MD, U.S.: Department of Health, Education, and Welfare; 1976.

11. Bandelow B. Panic and Agoraphobia Scale (PAS). Seattle, WA: Hogrefe & Huber; 1997.

12. Chambless DL, Caputo GC, Jasin SE, Gracely EJ, Williams C. The Mobility Inventory for Agoraphobia Behav Res Ther 1985; **23**: 35-44.

13. Oldfield RC. The assessment and analysis of handedness: the Edinburgh inventory. Neuropsychologia 1971; **9**: 97-113.

14. Posner MI, Petersen SE. The attention system of the human brain. Annu Rev Neurosci 1990; **13**: 25-42.

15. Eriksen BA, Eriksen CW. Effects of noise letters upon identification of a target letter in a non- search task. Perception and Psychophysics 1974; **16**: 143-9.

16. Neufang S, Geiger MJ, Homola GA, Mahr M, Akhrif A, Nowak J, et al. Modulation of prefrontal functioning in attention systems by NPSR1 gene variation. Neuroimage 2015; **114**: 199-206.

17. Fan J, McCandliss BD, Fossella J, Flombaum JI, Posner MI. The activation of attentional networks. Neuroimage 2005; **26**: 471-9.

18. Benjamini Y, Hochberg Y. Controlling the False Discovery Rate: A Practical and Powerful Approach to Multiple Testing. Journal of the Royal Statistical Society, Series B (Methodological) 1995; **57**: 289-300.

19. Richter J, Hamm AO, Pane-Farre CA, Gerlach AL, Gloster AT, Wittchen HU, et al. Dynamics of defensive reactivity in patients with panic disorder and agoraphobia: implications for the etiology of panic disorder. Biol Psychiatry 2012; **72**: 512-20.

20. Bouton ME, Mineka S, Barlow DH. A modern learning theory perspective on the etiology of panic disorder. Psychol Rev 2001; **108**: 4-32.

21. Pauli P, Marquardt C, Hartl L, Nutzinger DO, Holzl R, Strian F. Anxiety induced by cardiac perceptions in patients with panic attacks: a field study. Behav Res Ther 1991; **29**: 137-45.
